# Nanoimmunomodulation for cartilage repair in osteoarthritis: reprogramming the inflammatory joint microenvironment

**DOI:** 10.1016/j.ijpx.2026.100655

**Published:** 2026-08-28

**Authors:** Hanning Wang, Fei Wang, Sijia Han, Ling Li, Letong Liu, Zhan Wang

**Affiliations:** a Department of Orthopedics, The First Hospital of China Medical University, Shenyang, Liaoning Province 110001, China; b Department of Otolaryngology, The first Hospital of China Medical University, Shenyang, China; c Department of Oncology, Shengjing Hospital of China Medical University, Shenyang, Liaoning, China; d Department of Endocrinology, Shengjing Hospital of China Medical University, Shenyang, China

**Keywords:** Osteoarthritis, Nanoimmunomodulation, Cartilage repair, Joint microenvironment

## Abstract

Osteoarthritis is a whole-joint disease characterized by persistent inflammation, immune dysregulation, oxidative stress, cellular senescence, and impaired cartilage repair. Current treatments remain largely symptomatic, underscoring the need for disease-modifying strategies that can reshape the pathological joint microenvironment. Nanoimmunomodulation offers a promising approach by combining targeted intra-articular delivery, controlled release, biomimetic design, and immune reprogramming. In this Review, we discuss how nanoplatforms, including polymeric nanoparticles, lipid vesicles, inorganic nanozymes, extracellular vesicle-inspired systems, cell-membrane-coated nanoparticles, and hydrogel–nanoparticle composites, regulate synovial macrophage polarization, inflammasome activation, ROS accumulation, mitochondrial dysfunction, ferroptosis, cellular senescence, and cartilage matrix catabolism. We further propose a translational framework linking nanomaterial design to disease-stage-specific immune phenotypes, joint retention, cartilage penetration, safety, manufacturability, and clinically meaningful endpoints. While nanoimmunomodulation offers a compelling preclinical rationale for targeting multiple pathological drivers simultaneously, its translation into clinically validated disease-modifying therapy remains contingent upon overcoming significant hurdles in safety, manufacturing, and regulatory endpoint alignment. This review proposes a phenotype-driven framework to guide future clinical development rather than asserting imminent clinical efficacy.

## Graphical abstract

Osteoarthritis progression is driven by a pathological joint microenvironment characterized by synovial inflammation, oxidative stress, cellular senescence, ferroptosis, and impaired cartilage matrix homeostasis. Nano-immunomodulatory platforms enable targeted intra-articular delivery, immune reprogramming, redox regulation, and matrix repair, thereby shifting the OA joint from a catabolic state toward cartilage protection and disease modification.

**Figure:**
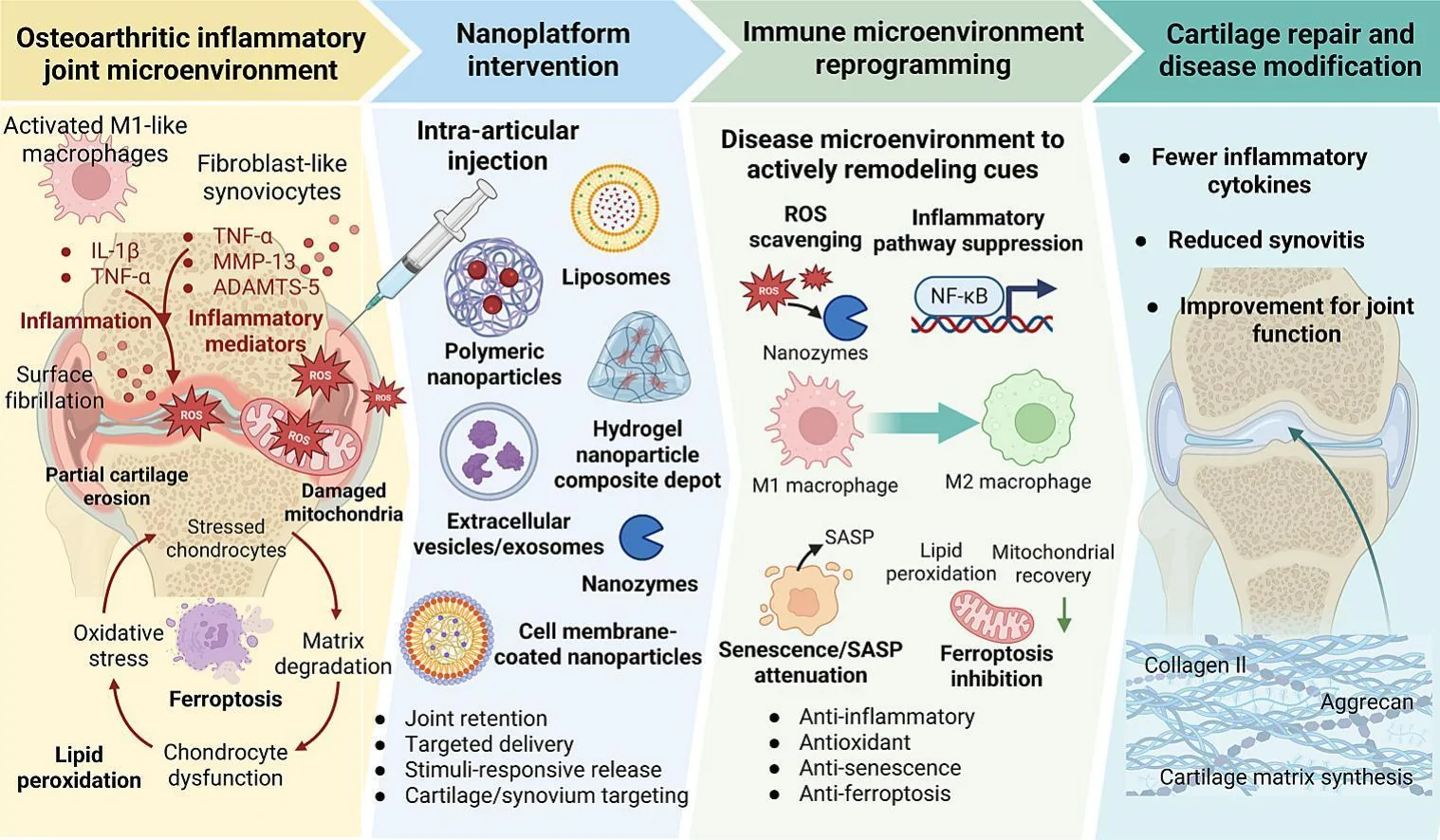

## 1 Introduction

Osteoarthritis (OA) is one of the most prevalent chronic joint diseases worldwide and a leading cause of pain, disability, impaired mobility, and reduced quality of life, particularly among older adults (Siddiq et al., 2024). With population aging, obesity, and increasing joint injury, the global burden of OA is expected to continue rising (GBD 2021 Osteoarthritis Collaborators, 2023). However, current treatments remain largely symptomatic. Non-steroidal anti-inflammatory drugs, intra-articular corticosteroids, and hyaluronic acid injections can provide temporary pain relief (Bannuru et al., 2019; Zhang et al., 2016a), but they do not halt cartilage degeneration or restore durable joint homeostasis (Di Francesco et al., 2022; Kou et al., 2019). Surgical interventions, such as total joint replacement, are generally reserved for end-stage disease and are not suitable for early disease modification (Oo, 2024). Therefore, effective disease-modifying OA therapies are urgently needed (Li et al., 2023). Another major barrier to OA therapy is disease heterogeneity. Patients with similar radiographic severity may differ markedly in inflammatory burden, pain mechanisms, metabolic status, subchondral bone changes, cartilage degeneration rate, and response to treatment. Increasing evidence supports distinct OA phenotypes or endotypes, including inflammatory, metabolic, mechanical, pain-dominant, senescence-associated, and subchondral bone-driven subgroups (Deveza et al., 2019). This heterogeneity highlights the need for mechanism-based and phenotype-guided therapeutic strategies rather than uniform symptom control.

In this context, nanoimmunomodulation has emerged as a promising strategy for OA treatment. Nanomaterials possess tunable size, surface charge, morphology, degradability, drug-loading capacity, and surface functionality, allowing them to improve intra-articular retention, enhance cartilage or synovial targeting, enable controlled or stimuli-responsive release, and reduce systemic exposure (Li et al., 2021; Yang et al., 2025). More importantly, nanoimmunomodulation can actively reprogram the pathological joint microenvironment by regulating macrophage polarization (Figueroa-Valdés et al., 2025), suppressing inflammatory signaling (Liang et al., 2025), scavenging ROS (Zuo et al., 2023), restoring mitochondrial function (Zhai et al., 2021), inhibiting ferroptosis (Xu et al., 2025b), attenuating cellular senescence (Lin et al., 2025), and promoting cartilage matrix anabolism (Chen et al., 2025a). Therefore, it offers a multifunctional therapeutic approach that is better aligned with the complex pathobiology of OA than conventional single-target interventions. Although nano-immunomodulation theoretically fits the multi-factor pathological characteristics of OA, it should be recognized that previous disease modifying osteoarthritis drug (DMOAD) candidate drugs targeting inflammatory mediators or matrix degrading enzymes have been terminated in Phase II/III clinical trials due to the lack of structural benefits (Van Spil et al., 2019). This suggests that the anti-inflammatory or restorative potential of nanomaterials alone is not sufficient to guarantee clinical success. We must draw lessons from previous trials in patient stratification and endpoint selection.

The understanding of OA has shifted from a simple “wear-and-tear” disorder to a complex whole-joint disease involving cartilage, synovium, subchondral bone, menisci, ligaments, infrapatellar fat pad, and periarticular tissues (Mathiessen and Conaghan, 2017; O'Neill and Felson, 2018; Chen et al., 2024a). Current investigations indicated that the unique physicochemical attributes of nanomaterials, specifically their superior catalytic activity, bio-enzyme-like reaction kinetics, and ability to regulate joint immunity, position them as a leading candidate for OA therapy (Deng et al., 2025a). Activated synovial macrophages, fibroblast-like synoviocytes, chondrocytes, and other joint-resident cells produce inflammatory cytokines, chemokines, reactive oxygen species (ROS), matrix-degrading enzymes, and senescence-associated secretory phenotype (SASP) factors, thereby forming a self-amplifying cycle of inflammation, cartilage destruction, and impaired repair (Mathiessen and Conaghan, 2017; Wijesinghe et al., 2024; Nanus et al., 2021; Damerau et al., 2024; Zeng et al., 2025). This multi-cellular and multi-pathway pathology partly explains why single-target therapies often show limited clinical efficacy. Growing evidence identifies macrophages as central mediators of OA pathophysiology, with the dysregulation of M1/M2 macrophage polarization promotes synovial inflammation, cartilage degradation, and impaired repair mechanisms, reaching reprogramming rates of up to 88.5% (Codispoti et al., 2026; Afshari et al., 2026). Therefore, targeting this immunological imbalance offers a promising avenue for developing disease-modifying therapies for OA. While chondrocytes and synovial fibroblasts are fundamental effectors of cartilage degradation and synovial pathology, this review specifically focuses on nanoimmunomodulation strategies that target the immune microenvironment as the upstream driver of OA progression. We emphasize macrophage reprogramming not to exclude other cell types, but because restoring immune homeostasis is increasingly recognized as a prerequisite for durable cartilage repair and for breaking the vicious cycle of inflammation-senescence-catabolism involving all joint-resident cells (Cheng et al., 2026).

This review not only summarizes recent advances in nanoimmunomodulation for cartilage repair in OA, but also proposes specific nanoimmunomodulatory strategies matched with the specific molecular driver phenotypes of patients. A precisive perspective that directly links spatial transcriptomics, biomarker stratification and nanoplatform design is lacking in most existing OA reviews. We first discuss the inflammatory and immune-metabolic features of the osteoarthritic microenvironment, then review major nanoplatforms and their immune regulatory mechanisms, followed by their cartilage repair effects and key translational challenges. Finally, we propose a disease-phenotype-driven framework for nanomaterial design, aiming to advance nanoimmunomodulation from empirical preclinical efficacy toward personalized, clinically translatable, and disease-modifying OA therapy.

## 2 Inflammatory microenvironment

The pathogenesis of OA is intricately linked to a complex and dynamic inflammatory microenvironment within the joint, which actively drives cartilage degradation and impedes repair processes (Berenbaum, 2012; South et al., 2025). This microenvironment is characterized by the aberrant activation and interplay of various cellular and molecular components, including synovial macrophages (Zhang et al., 2018), pro-inflammatory cytokines (Sanchez-Lopez et al., 2022), ROS (Zhang et al., 2023a), and the accumulation of senescent cells (Swahn et al., 2022), all contributing to a vicious cycle of tissue damage. Understanding these underlying mechanisms is paramount for developing effective therapeutic interventions, particularly those leveraging nanoimmunomodulation to reprogram this pathological milieu.

### 2.1 Synovial macrophages and inflammation

**Fig. 1:**
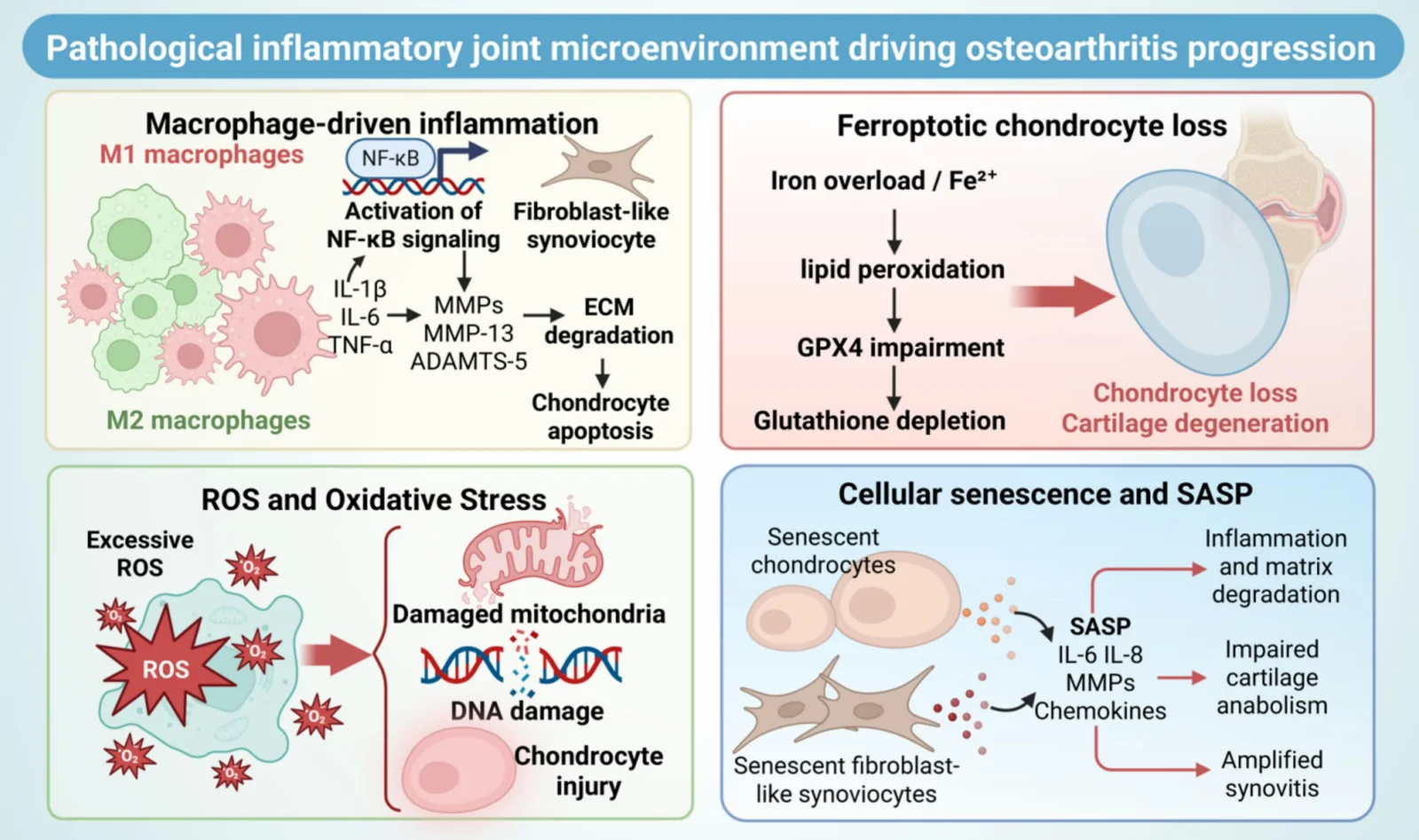
Pathological inflammatory joint microenvironment in osteoarthritis. The osteoarthritic joint microenvironment contains activated synovial macrophages, pro-inflammatory cytokines, excessive ROS, ferroptotic chondrocytes, and senescent cells with SASP release. These interacting pathological factors promote extracellular matrix degradation, chondrocyte dysfunction, synovial inflammation, and impaired cartilage repair.

Synovial macrophages play a pivotal role in orchestrating the inflammatory response in OA, exhibiting remarkable plasticity to polarize into distinct phenotypes that either promote (M1-like) or resolve (M2-like) inflammation (Chang and Tang, 2024). In the OA joint, a predominant shift toward the pro-inflammatory M1 phenotype is observed, characterized by the secretion of a plethora of catabolic enzymes and inflammatory mediators such as interleukin-1 beta (IL-1β), tumor necrosis factor-alpha (TNF-α), and matrix metalloproteinases (MMPs) (Chang and Tang, 2024; Huang et al., 2024). This M1-driven inflammation directly contributes to chondrocyte apoptosis, extracellular matrix (ECM) degradation, and overall joint destruction (Fang et al., 2024). For instance, the inflammatory microenvironment, particularly when rich in IL-1β, can repurpose BMP-2 signaling to drive pathological osteophyte formation rather than cartilage repair, highlighting the detrimental impact of sustained inflammation (Yu et al., 2026). The activation of nuclear factor-kappa B (NF-κB) signaling pathways in chondrocytes and synovial cells, often triggered by IL-1β, is a central event in OA pathogenesis, leading to increased expression of catabolic factors like MMP-13 and ADAMTS-5 (Murahashi et al., 2018; Li et al., 2025a). Furthermore, a detrimental feedback loop exists between senescent cells and M1 macrophages in synovitis, where senescent cells secrete a SASP that promotes M1 polarization, thereby exacerbating inflammation and perpetuating the disease (Zhang et al., 2026a). This intricate cellular crosstalk, involving immune cells, fibroblasts, and nociceptors in the inflamed synovium, is also a significant driver of nociceptive joint pain in OA, further emphasizing the need for immunomodulatory strategies (Wijesinghe et al., 2024). Therefore, reprogramming synovial macrophages from M1 to M2 phenotype is a critical therapeutic target for nanoimmunomodulation (Fig. 1).

### 2.2 ROS and oxidative stress

The OA joint microenvironment is characterized by a profound imbalance between the production and scavenging of ROS, leading to chronic oxidative stress. Chondrocytes, under inflammatory conditions, generate excessive ROS, which directly damages cellular components, including DNA, proteins, and lipids, contributing to mitochondrial dysfunction and chondrocyte apoptosis (Singh et al., 2025; Lu et al., 2023; Yang et al., 2026). This oxidative stress also activates various signaling pathways that promote inflammation and catabolism, further accelerating cartilage degradation (Zheng et al., 2026). For example, ROS accumulation is a key driver of cartilage degradation and OA progression, making ROS scavenging a critical therapeutic target (Zheng et al., 2026). The hypoxic microenvironment often found in OA joints further exacerbates oxidative stress, as reoxygenation can lead to a burst of ROS production, creating a vicious cycle of damage (Wang et al., 2025c). This persistent oxidative assault not only compromises chondrocyte viability but also contributes to the overall inflammatory burden, creating a self-perpetuating cycle of damage. Therefore, strategies aimed at restoring redox homeostasis are crucial for protecting chondrocytes and preserving cartilage integrity (Fig. 1).

### 2.3 Ferroptosis

Beyond apoptosis and necrosis, ferroptosis, an iron-dependent form of regulated cell death characterized by the accumulation of lipid peroxides, has emerged as a significant contributor to chondrocyte loss in OA (Xu et al., 2025b). Excessive intracellular iron accumulation, often exacerbated by oxidative stress, can trigger ferroptosis in chondrocytes, leading to their demise and subsequent cartilage degradation (Xu et al., 2025b). This distinct cell death pathway is driven by an imbalance in iron metabolism and antioxidant defense systems, particularly the glutathione peroxidase 4 (GPX4) pathway, which normally detoxifies lipid peroxides. The depletion of glutathione, a substrate for GPX4, or direct inhibition of GPX4 activity, renders cells vulnerable to ferroptosis (Fig. 1). Understanding the molecular triggers of ferroptosis in OA, such as iron overload and lipid peroxidation, provides novel therapeutic avenues for preserving chondrocyte viability and mitigating OA progression (Yue et al., 2025).

### 2.4 Cellular senescence

Cellular senescence, a state of irreversible cell cycle arrest accompanied by a pro-inflammatory senescence-associated secretory phenotypes (SASPs), is increasingly recognized as a fundamental driver of OA pathogenesis (Han et al., 2024). Senescent chondrocytes, synovial fibroblasts, and other joint cells accumulate in OA joints with aging and disease progression (Cao et al., 2023; He et al., 2026). These senescent cells secrete a cocktail of inflammatory cytokines, chemokines, and matrix-degrading enzymes, which not only perpetuate inflammation but also induce senescence in neighboring healthy cells, creating a self-amplifying cycle of damage (Zhang et al., 2026a; Han et al., 2024). The SASP contributes to ECM degradation, inhibits chondrocyte anabolism, and promotes M1 macrophage polarization, thereby accelerating OA progression. This vicious cycle highlights the importance of targeting senescent cells (senolysis) or modulating their SASP to reprogram the joint microenvironment and promote cartilage repair (Zhang et al., 2026a; Shen et al., 2025). The metabolic dysfunction associated with senescent cells, including mitochondrial impairment, further contributes to the overall pathological state, making mitochondrial rejuvenation a promising strategy (Fig. 1).

In summary, the OA joint microenvironment is a complex interplay of immune cells, inflammatory mediators, oxidative stress, and senescent cells, all contributing to a chronic, destructive process (Feng et al., 2026, Li et al., 2026b, Liao et al., 2024a, Liao et al., 2025b, Liao et al., 2025a). Effective therapeutic strategies must therefore adopt a multi-pronged approach to reprogram this pathological environment, shifting it from a catabolic to an anabolic state. Nanoimmunomodulation, with its ability to precisely deliver therapeutic agents and modulate cellular functions, offers a unique opportunity to address these multifaceted challenges by simultaneously targeting multiple pathological pathways.

## 3 Nanoplatforms for microenvironment reprogramming

**Fig. 2:**
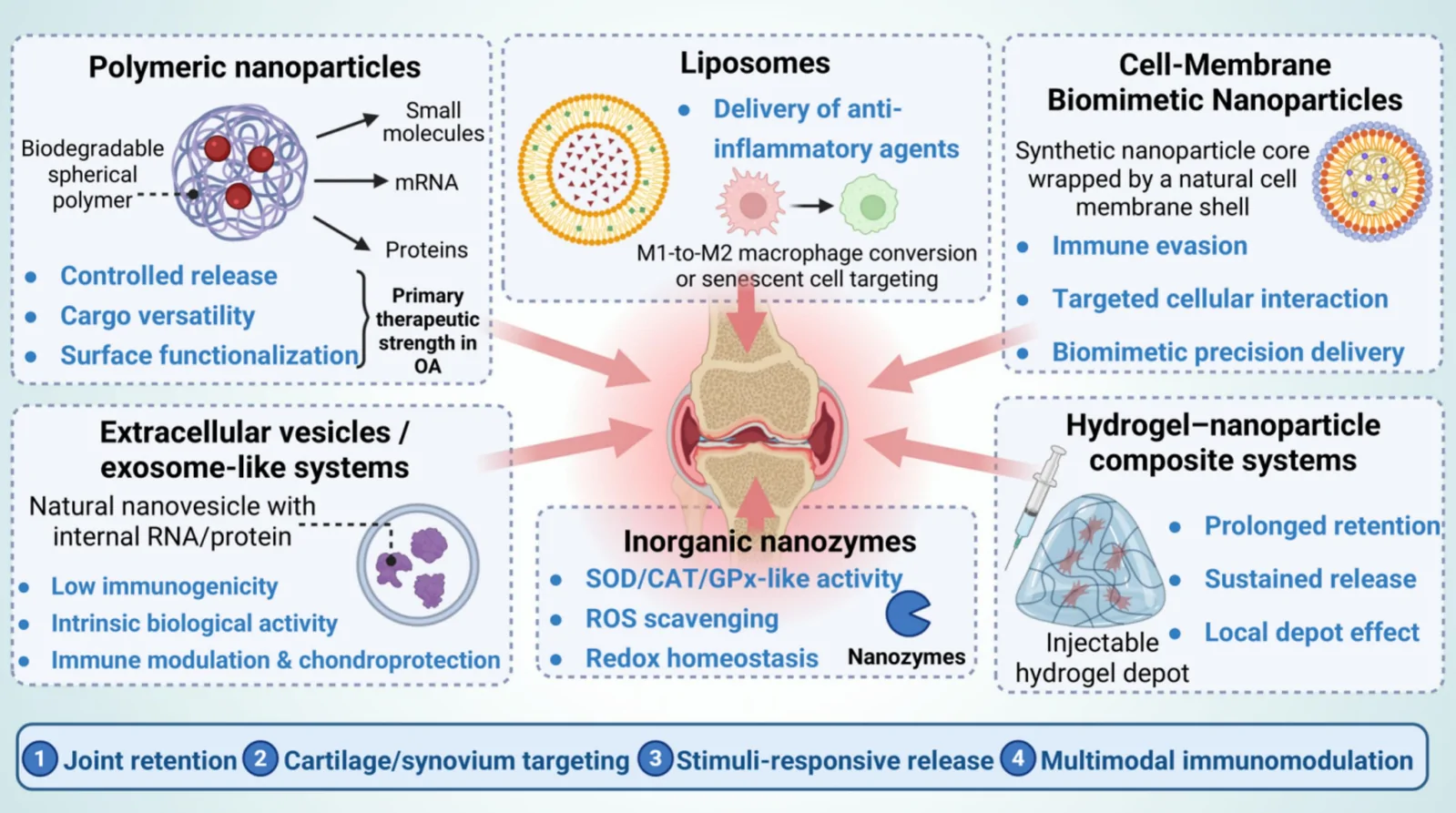
Nanoplatform toolbox for osteoarthritis immunomodulation. Polymeric nanoparticles, liposomes, nanozymes, extracellular vesicles, membrane-coated nanoparticles, and hydrogel–nanoparticle composites provide complementary strategies for osteoarthritis therapy. These platforms improve joint retention, cartilage or synovium targeting, controlled release, ROS clearance, and immune microenvironment reprogramming.

The intricate and hostile microenvironment of the osteoarthritic joint present significant challenges for conventional drug delivery, including rapid clearance of intra-articularly injected agents, poor penetration into dense cartilage tissue, and non-specific distribution leading to systemic side effects (Di Francesco et al., 2022; Kou et al., 2019; Brown et al., 2019). Nanoplatforms, owing to their tunable size, surface properties, and ability to encapsulate diverse therapeutic cargoes, offer a transformative solution to overcome these barriers and enable targeted, sustained, and effective reprogramming of the inflammatory joint microenvironment. These platforms are designed to enhance intra-articular retention, facilitate cartilage penetration, and precisely modulate immune responses and cellular processes (Fig. 2). Each class of nanoplatform brings distinct advantages and disadvantages to the complex task of OA therapy, as summarized in Table 1.

**Table 1:** Comparison of nanoplatforms for osteoarthritis therapy.

Nanoplatform	Core idea	Advantages	Disadvantages	Literature
Polymer Nanoparticles	Biodegradable polymers encapsulate drugs/genes for controlled release.	High versatility in cargo, tunable release kinetics, surface functionalization for targeting, good biocompatibility.	Potential for burst release, limited cartilage penetration without modification, batch-to-batch variability.	(Mancipe Castro et al., 2020; Aini et al., 2016; Sorasitthiyanukarn et al., 2025)
Liposomes	Self-assembling lipid bilayer vesicles mimic biological membranes.	Excellent biocompatibility, low immunogenicity, encapsulate both hydrophilic/hydrophobic drugs, reduced systemic toxicity.	Relatively low stability in vivo, rapid clearance without modification, limited intrinsic targeting.	(Zhang et al., 2026a)
Inorganic Nanoenzymes (Nanozymes)	Nanomaterials with intrinsic enzyme-like catalytic activities (e.g., SOD, CAT).	High stability, cost-effective production, potent ROS scavenging, multi-enzyme mimicry.	Potential long-term toxicity/accumulation, limited biodegradability for some types, lack of intrinsic targeting without functionalization.	(Singh et al., 2025; Lu et al., 2023; Zheng et al., 2026)
Exosome-like Systems (EVs)	Naturally occurring nanovesicles for intercellular communication.	Inherent biocompatibility, low immunogenicity, natural targeting, complex cargo (proteins, lipids, nucleic acids), direct immunomodulatory effects.	Challenging for large-scale production, purification and standardization issues, cargo loading efficiency, rapid clearance without modification.	(Chen et al., 2025a; Cao et al., 2023; Ge et al., 2026)
Cell-Membrane Biomimetic Nanoparticles	Synthetic core coated with natural cell membranes (e.g., macrophage, RBC).	Inherit surface properties of source cells (targeting, immune evasion), enhanced biocompatibility, reduced immunogenicity.	Complex fabrication, potential for membrane degradation, limited cargo capacity compared to synthetic polymers.	(Yue et al., 2025)
Hydrogel-Nano Composite Platforms	Nanoparticles embedded within a hydrogel matrix.	Sustained intra-articular retention, localized delivery, protection of nanoparticles, tunable mechanical properties, synergistic effects.	Limited cartilage penetration of the bulk hydrogel, potential for swelling/degradation issues, complex multi-component system.	(Chen et al., 2025a; Holyoak et al., 2019; Yang et al., 2024)

### 3.1 Polymer nanoparticles

Polymer nanoparticles are highly versatile nanocarriers, widely explored for OA therapy due to their biodegradability, biocompatibility, and ability to encapsulate a broad range of therapeutic molecules, from small drugs to nucleic acids and proteins (Li et al., 2025b, Liao et al., 2024b, Liao et al., 2026c, Liao et al., 2026a). Their design allows for controlled release kinetics and surface functionalization for targeted delivery. For instance, poly (lactic-*co*-glycolic acid) (PLGA) nanoparticles have been integrated into microgels to achieve sustained release of drugs within the joint space, addressing the issue of rapid clearance (Mancipe Castro et al., 2020). The advantage here lies in the ability to fine-tune degradation rates and drug release profiles, offering prolonged therapeutic windows. Similarly, polymer-based nanomicelles, such as PEG-polyamino acid nanomicelles, have been successfully used to deliver messenger RNA (mRNA) encoding cartilage-anabolic transcription factors like RUNX1, demonstrating the potential for gene therapy in OA (Aini et al., 2016). This highlights their capacity to deliver sensitive biological molecules, which is critical for reprogramming cellular functions. Chitosan (CS) and its derivatives, natural biopolymers with low toxicity and good biocompatibility, are increasingly utilized in nanomedicine for OA. CS-based nanoparticles can modulate inflammation, oxidative stress, and promote cartilage repair (Zhang et al., 2026b). For example, fucoxanthin encapsulated in alginate/chitosan nanoparticles (FX-Alg/CS-NPs) showed enhanced anti-inflammatory activity in OA cell models by suppressing key inflammatory mediators and MAPK signaling, highlighting the advantage of nanoencapsulation for improving drug efficacy and bioavailability (Sorasitthiyanukarn et al., 2025). The ability of polymer nanoparticles to be engineered for specific targeting, such as cartilage-targeting polymers, further enhances their therapeutic potential by improving localization and reducing off-target effects (Cao et al., 2023). However, achieving deep cartilage penetration with larger polymer nanoparticles remains a challenge without specific surface modifications.

### 3.2 Liposomes

Liposomes are self-assembling lipid bilayer vesicles that mimic biological membranes, offering excellent biocompatibility and the ability to encapsulate both hydrophilic and hydrophobic drugs. Their biomimetic nature allows for reduced immunogenicity and enhanced cellular uptake. In the context of OA, liposomes have been explored for targeted delivery of anti-inflammatory agents and senolytics. For example, liposomes have been designed as “dual nano-switches” to deliver senolytics for clearing senescent cells and exosomes for converting pro-inflammatory M1 macrophages to anti-inflammatory M2 macrophages, thereby disrupting the SASP-M1 macrophage feedback loop in synovitis (Zhang et al., 2026a). This approach demonstrates the capacity of liposomes to carry complex therapeutic cargoes and achieve multi-modal immunomodulation, leveraging their ability to protect sensitive molecules and facilitate their cellular entry. While generally stable, liposomes can be susceptible to degradation by phospholipases in biological environments and may require surface modifications (e.g., PEGylation) to improve their stability and circulation time, particularly for intra-articular applications where rapid clearance is a concern.

### 3.3 Inorganic nanoenzymes (nanozymes)

Inorganic nanoenzymes, or nanozymes, are nanomaterials possessing intrinsic enzyme-like catalytic activities, such as superoxide dismutase (SOD), catalase (CAT), and glutathione peroxidase (GPx) mimics. These properties make them highly effective in scavenging ROS and mitigating oxidative stress, a key pathological feature of OA. A significant advantage of nanozymes over natural enzymes is their superior stability under harsh physiological conditions and lower production costs. Mesoporous ceria nanoparticles, for instance, have been functionalized (mCNP-G) to scavenge both ROS and cell-free DNA (cfDNA), downregulating pro-inflammatory signaling and showing high affinity for cartilage, offering a “push-and-pull” approach to OA therapy (Singh et al., 2025). Black phosphorus nanosheets (BPNSs) have been developed as dual-functional nanomedicines that eliminate intracellular ROS, protect cartilage, and promote osteogenesis, demonstrating their ability to address multiple facets of OA pathology (Lu et al., 2023). Similarly, bioinspired bimetallic copper‑manganese zeolitic imidazolate framework (CuMn-ZIF) nanozymes exhibit potent CAT and SOD-mimetic activities, effectively scavenging H2O2 and superoxide anions, rebalancing redox status, and restoring autophagic flux in OA chondrocytes (Zheng et al., 2026). Molybdenum disulfide (MoS2) nanosheets have also been utilized as near-infrared (NIR) light-responsive carriers for controlled release of dexamethasone, combining ROS scavenging with anti-inflammatory drug delivery (Zhao et al., 2019). The primary challenge with nanozymes lies in ensuring their long-term biodegradability and avoiding potential accumulation-related toxicity, especially with repeated dosing.

### 3.4 Exosome-like systems

Extracellular vesicles (EVs), particularly exosomes, are naturally occurring nanovesicles secreted by cells that play a crucial role in intercellular communication by transferring proteins, lipids, and nucleic acids. Their inherent biocompatibility, low immunogenicity, and ability to cross biological barriers make them attractive nanocarriers for OA therapy (Luan et al., 2017; Ehab et al., 2025). Mesenchymal stem cell (MSC)-derived exosomes (MSC-Exos) have shown significant promise due to their chondroprotective, anti-inflammatory, and immunomodulatory properties (Kim et al., 2020; Sun et al., 2025). A key advantage is their intrinsic biological activity; they don't just deliver cargo but also exert therapeutic effects through their native components. For example, exosomes derived from human umbilical cord mesenchymal stem cells (HucMSCs), especially when primed in a pro-inflammatory environment (IL-1β-induced exosomes or C-Exos), exhibit enhanced anti-inflammatory properties, promote chondrocyte functionality, and induce M2 macrophage polarization (Chen et al., 2025a). These C-Exos, when encapsulated in hyaluronic acid hydrogel microspheres, further improve the inflammatory microenvironment and cartilage regeneration (Chen et al., 2025a). Similarly, chondrocyte-targeting exosomes from umbilical cord-derived MSCs have been used to rejuvenate aging chondrocytes, with enhanced retention achieved by encapsulating them in hyaluronic acid microgels (Cao et al., 2023). Beyond MSCs, engineered M2 macrophage-derived extracellular vesicles (Cur@M2-EVs) encapsulating curcumin have been shown to reprogram mitochondrial metabolism, scavenge ROS, and alleviate temporomandibular joint cartilage degeneration, demonstrating the potential of immune cell-derived EVs for targeted immunomodulation (Ge et al., 2026). M2-sEVs have also been shown to alleviate OA pain by regulating synovial macrophage NGF expression via the NOTCH pathway (Liu et al., 2026). Despite their promise, challenges include scalable and standardized manufacturing, purification, and efficient cargo loading for therapeutic enhancement (Ehab et al., 2025; Wu et al., 2026; Zhao et al., 2026).

### 3.5 Cell-membrane biomimetic nanoparticles

Cell-membrane biomimetic nanoparticles involve coating synthetic nanoparticles with natural cell membranes (e.g., red blood cell membranes, macrophage membranes). This strategy endows the synthetic core with the surface properties of the source cell, including specific targeting ligands, immune evasion mechanisms, and reduced immunogenicity. For OA, macrophage membrane-coated nanoparticles are particularly relevant for immune reprogramming due to their ability to interact specifically with immune cells and evade immune clearance. For instance, a kartogenin (KGN)-loaded biomimetic system, CM@Lipos-KGN, utilizes cell membrane coating to mitigate cartilage degeneration and promote regeneration by suppressing inflammation and chondrocyte ferroptosis, and enhancing BMSC chondrogenic differentiation (Yue et al., 2025). This biomimetic approach allows for targeted interaction with immune cells and specific tissues within the joint, enhancing therapeutic precision while minimizing off-target effects. The primary challenge lies in the complex and reproducible fabrication of these systems, ensuring the integrity and functionality of the membrane coating.

### 3.6 Hydrogel-nano composite platforms

Hydrogel-nano composite platforms represent a synergistic approach, combining the advantages of injectable hydrogels for sustained intra-articular retention and localized delivery with the specific functionalities of embedded nanoparticles. Hydrogels, often composed of biocompatible polymers like hyaluronic acid, PEG, or chitosan, can form a three-dimensional network that acts as a depot, slowly releasing encapsulated nanoparticles or drugs over extended periods. This addresses the critical issue of rapid clearance from the joint space. For example, microgels containing PLGA nanoparticles have been used for sustained drug release (Mancipe Castro et al., 2020). Similarly, hyaluronic acid hydrogel microspheres have been employed to encapsulate IL-1β-primed exosomes, significantly improving their retention and efficacy in cartilage regeneration (Chen et al., 2025a). Injectable hydrogels, such as PEG-4MAL hydrogels, can maintain mechanical properties and release therapeutic payloads over extended periods, protecting the knee joint from cartilage degradation (Holyoak et al., 2019). The hydrogel matrix not only prolongs residence time but also protects sensitive nanoparticles from degradation in the harsh joint environment. Furthermore, the tunable mechanical properties of hydrogels can be designed to mimic native tissue, providing a supportive scaffold for repair. However, the bulk nature of hydrogels can limit their penetration into dense cartilage, often requiring the embedded nanoparticles to be designed for cartilage penetration after release. Magnetic polysaccharide hydrogel particles, by encapsulating magnetic nanoparticles and diclofenac sodium, demonstrated synergistic effects in alleviating OA symptoms and promoting cartilage repair, showcasing the potential for multi-modal therapy (Yang et al., 2024).

In summary, the diverse array of nanoplatforms provides an unprecedented toolkit for reprogramming the inflammatory joint microenvironment in OA. Each platform offers unique advantages in terms of cargo versatility, targeting capabilities, release kinetics, and immunomodulatory potential, paving the way for highly effective and personalized OA therapies. The strategic combination of these platforms, such as nanoparticles within hydrogels or biomimetic coatings on synthetic cores, further expands the therapeutic possibilities, allowing for multi-pronged attacks on OA pathology.

## 4 Immune regulatory mechanisms

**Fig. 3:**
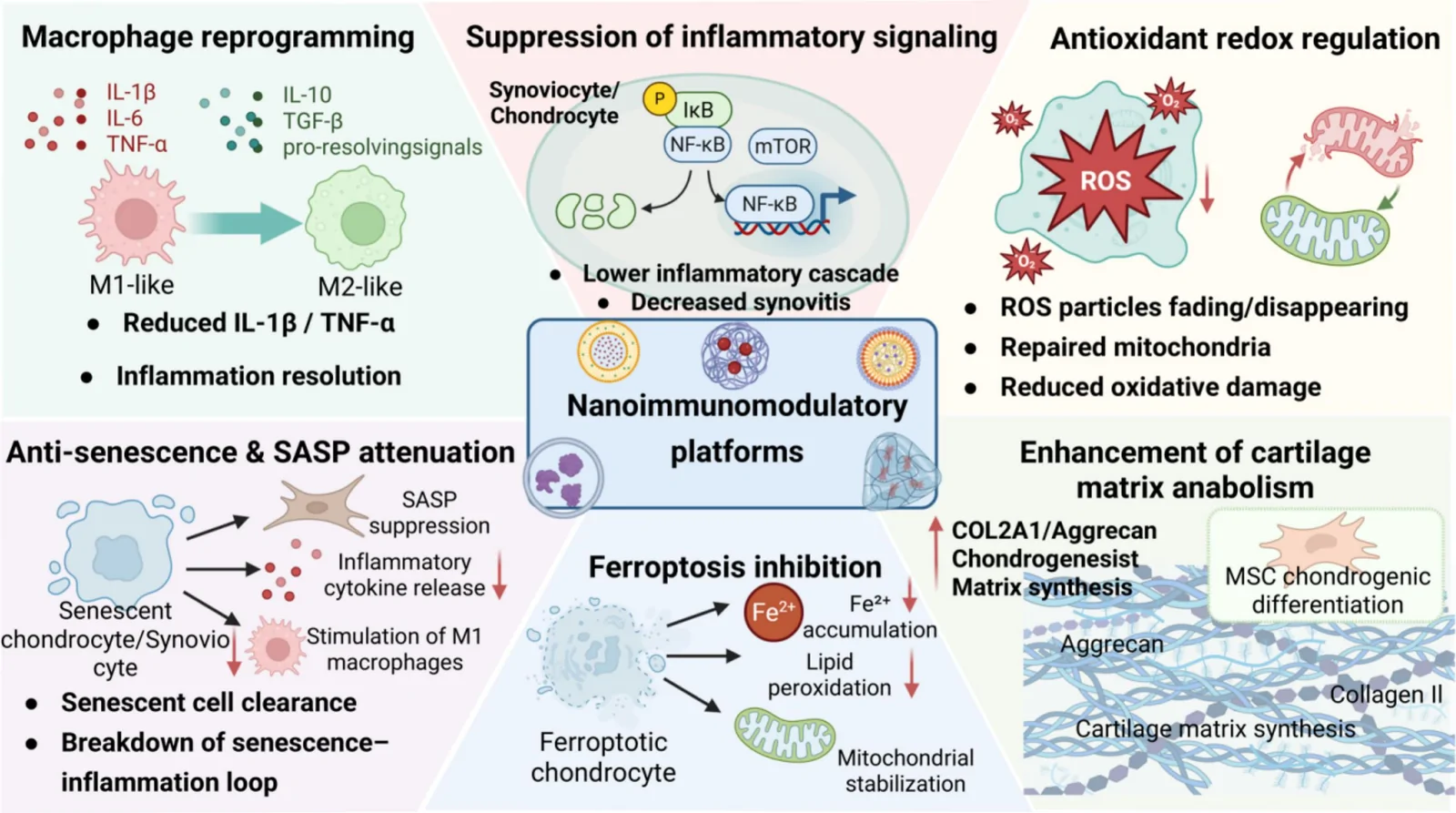
Mechanistic landscape of nano-immunomodulation in osteoarthritis. Nano-immunomodulation regulates osteoarthritis through coordinated mechanisms including M1-to-M2 macrophage polarization, inflammatory pathway suppression, ROS scavenging, SASP attenuation, ferroptosis inhibition, and cartilage matrix synthesis. Together, these mechanisms convert the osteoarthritic joint from a chronic catabolic and inflammatory niche into a more reparative microenvironment.

Nanoimmunomodulation for cartilage repair in OA operates through a sophisticated array of immune regulatory mechanisms, leveraging the unique properties of nanoplatforms to precisely target and reprogram the pathological joint microenvironment (Moulin et al., 2025; Zhang et al., 2023b; Yan et al., 2026a). These mechanisms include macrophage reprogramming, inflammation suppression, antioxidant effects, anti-aging properties, anti-ferroptosis actions, and enhanced synthesis of cartilage matrix components, all contributing to a shift from a catabolic to an anabolic state within the joint (Fig. 3).

### 4.1 Macrophage reprogramming

A cornerstone of nanoimmunomodulation is the ability to reprogram synovial macrophages, shifting their phenotype from pro-inflammatory M1 to anti-inflammatory and pro-resolving M2. This phenotypic switch is crucial for resolving chronic inflammation and promoting tissue repair. Nanoplatforms achieve this through various strategies: (1) Targeted delivery of immunomodulatory agents: Nanoparticles can be engineered to specifically target M1 macrophages, delivering agents that promote their M2 repolarization. For instance, IgG-conjugated bilirubin/JPH203 nanoparticles (IgG/BRJ) are designed to be recognized and engulfed by M1 macrophages. Once internalized, bilirubin and JPH203 are released, which scavenge ROS, inhibit NF-κB and mTOR pathways, and ultimately enhance the M2/M1 ratio, thereby suppressing inflammation and promoting cartilage protection (Huang et al., 2024). This highlights the precision of targeting specific immune cell subsets. (2) Delivery of specific nucleic acids: Gene silencing or activation via nucleic acid delivery is a powerful tool for macrophage reprogramming. AHK-CaP/siCA9 NPs, for instance, deliver siRNA targeting CA9, which downregulates CA9 mRNA to repolarize M1 macrophages to M2, improving the inflammatory environment and promoting chondrogenesis (Cui et al., 2024). Similarly, dual-modular hydrogel microparticles (dmHMPs) are engineered to modulate the inflammatory microenvironment by promoting anti-inflammatory macrophage polarization, which reduces hypertrophic cartilage formation and preserves cartilage integrity (Chen et al., 2025b). This demonstrates the potential to alter gene expression profiles to drive phenotypic shifts. (3) Utilizing extracellular vesicles: EVs, particularly those derived from M2 macrophages or MSCs, inherently possess immunomodulatory properties that promote M2 polarization and alleviate inflammation. Curcumin-encapsulated M2-EVs (Cur@M2-EVs) further enhance this effect by scavenging ROS and restoring mitochondrial function, leading to reduced inflammation and matrix degradation (Ge et al., 2026). M2-sEVs have also been shown to reduce OA pain by inhibiting synovial macrophage NGF expression via the Notch pathway, highlighting their broader immunomodulatory effects beyond just inflammation (Liu et al., 2026). Platelet-rich plasma (PRP)-derived microRNA let-7a-5p, delivered via nanocarriers, can regulate macrophage polarization by inhibiting M1 and promoting M2 phenotypes, thereby improving the inflammatory microenvironment in OA (Li et al., 2026a). TREM2, delivered via nanoplatforms, can also promote macrophage polarization from M1 to M2, suppressing OA progression through the NF-κB/CXCL3 axis (Fang et al., 2024). These examples underscore the versatility of nanoplatforms in delivering diverse molecular signals to orchestrate macrophage reprogramming.

### 4.2 Inflammation suppression

Beyond direct macrophage reprogramming, nanoplatforms actively suppress inflammation through various mechanisms, often by targeting key inflammatory mediators and signaling pathways. This includes inhibiting the production of pro-inflammatory cytokines, reducing the activation of inflammasomes, and blocking downstream signaling cascades. For instance, the IgG/BRJ nanoparticles, by inhibiting NF-κB and mTOR pathways, directly suppress the inflammatory response in M1 macrophages (Huang et al., 2024). Similarly, the ultra-long-term anti-inflammatory polyphenol capsule, by remodeling the microenvironment, achieved a single-dose therapeutic effect in mouse and dog OA models, demonstrating potent and sustained inflammation suppression (Wei et al., 2024). This sustained release of anti-inflammatory agents, facilitated by nanocarriers, is crucial for overcoming the chronic nature of OA inflammation. Furthermore, the ROS-responsive nanomedicine (G4-TBP NPs-FN) exhibited ROS scavenging, hypoxia attenuation, and M2 macrophage polarization, collectively reducing joint pathology and inhibiting synovitis (Zhan et al., 2024). By simultaneously addressing oxidative stress and immune cell polarization, these nanoplatforms provide a comprehensive approach to inflammation suppression.

### 4.3 Antioxidant effects

Oxidative stress, driven by excessive ROS, is a major contributor to OA pathology. Nanoplatforms with intrinsic antioxidant properties or those delivering antioxidant agents effectively scavenge ROS and restore redox homeostasis, thereby protecting chondrocytes and mitigating inflammation. (1) Nanozymes as ROS scavengers: Inorganic nanozymes are particularly adept at this. Mesoporous ceria nanoparticles (mCNP-G) act as potent ROS scavengers, reducing oxidative stress in osteoarthritic cartilage by mimicking superoxide dismutase and catalase activities (Singh et al., 2025). Black phosphorus nanosheets (BPNSs) eliminate intracellular ROS, protecting cartilage and promoting osteogenesis (Lu et al., 2023). The CuMn-ZIF nanozyme, with its CAT and SOD-mimetic activities, rebalances redox status and suppresses PI3K–AKT–mTOR signaling in OA chondrocytes, demonstrating a direct link between ROS scavenging and anti-catabolic effects (Zheng et al., 2026). Selenium-doped layered double hydroxide nanocomposites deliver mitochondrial-targeted ROS scavengers, enhancing OA therapy via inflammation suppression and macrophage metabolism reprogramming, highlighting the importance of subcellular targeting for antioxidant effects (Fang et al., 2026). (2) Antioxidant drug delivery: Nanocarriers can also enhance the delivery and efficacy of conventional antioxidant drugs. Polyphenol capsules enhance cellular uptake and scavenge reactive oxygen and nitrogen species, reducing inflammatory markers and inhibiting the p38 MAPK pathway in the OA microenvironment (Wei et al., 2024). Oxygen-releasing biomaterials, by increasing local oxygen partial pressure, can suppress oxidative stress and mitigate the hypoxic joint microenvironment, thereby indirectly reducing ROS production and damage (Wang et al., 2025c). These strategies collectively aim to restore the delicate redox balance essential for chondrocyte health and cartilage integrity.

### 4.4 Anti-aging properties (senolysis)

Cellular senescence is increasingly recognized as a central driver of OA progression rather than a passive consequence of tissue aging. In the osteoarthritic joint, senescent chondrocytes, synovial fibroblasts, macrophages, and other resident cells accumulate progressively and acquire a SASP, characterized by the persistent release of pro-inflammatory cytokines, chemokines, matrix-degrading enzymes, and catabolic mediators. This SASP not only directly accelerates ECM degradation and suppresses cartilage anabolism but also amplifies synovial inflammation by promoting M1-like macrophage polarization, thereby forming a self-perpetuating feedback loop between senescent cells and inflammatory immune cells. Nanoimmunomodulatory strategies provide a promising means to interrupt this pathological cycle by either selectively eliminating senescent cells or attenuating their pro-inflammatory secretory activity. For example, liposome-based “dual nano-switches” have been designed to deliver senolytic agents for targeted clearance of senescent cells while simultaneously modulating macrophage polarization, thereby disrupting the SASP–M1 macrophage feedback loop and restoring joint immune homeostasis (Zhang et al., 2026a). Such targeted senolytic delivery is particularly important because systemic or non-selective elimination of senescent cells may damage normal cells involved in tissue repair and homeostatic maintenance (Xu et al., 2026). Beyond senescent-cell clearance, nanoplatforms can also act through senomorphic or metabolic rejuvenation mechanisms. Since senescent chondrocytes commonly exhibit mitochondrial dysfunction, impaired respiratory chain activity, redox imbalance, and decreased NAD^+^ metabolism, restoring mitochondrial fitness represents an important anti-senescence strategy. A mitochondria-targeted NAD^+^/O₂ co-delivery interpenetrating network hydrogel, for instance, can improve respiratory chain function, enhance antioxidant enzyme activity, increase the NAD^+^/NADH ratio, reduce oxidative stress, and suppress senescence-related factors, thereby alleviating chondrocyte senescence and slowing OA progression (Shen et al., 2025). Therefore, anti-senescence nanomedicine should not be limited to senescent-cell removal, but may also include modulation of SASP activity, mitochondrial restoration, redox rebalancing, and metabolic reprogramming. By targeting both the cellular and microenvironmental consequences of senescence, these nanoplatforms offer a more comprehensive strategy for breaking the inflammation–senescence–matrix degradation cycle and promoting durable cartilage protection in OA.

### 4.5 Anti-ferroptotic actions

Ferroptosis has emerged as an important mechanism of chondrocyte loss in OA, linking iron overload, oxidative stress, lipid peroxidation, and cartilage degeneration. Unlike apoptosis, ferroptosis is driven by iron-dependent accumulation of lipid peroxides and failure of cellular antioxidant defenses, making it particularly relevant to the oxidative and inflammatory microenvironment of OA. Nanoimmunomodulatory platforms can protect chondrocytes from ferroptotic injury by regulating iron homeostasis and suppressing lipid peroxidation. For example, β-diketone-functionalized microspheres can chelate reactive iron within peroxidative joint microenvironments, downregulate TfR1 expression, reduce mitochondrial iron uptake, and restore mitochondrial iron balance, thereby inhibiting ferroptosis and slowing OA progression (Xu et al., 2025b). Similarly, kartogenin-loaded biomimetic nanomedicine, such as CM@Lipos-KGN, may suppress chondrocyte ferroptosis while simultaneously reducing inflammation and promoting cartilage regeneration, partly through preservation of redox homeostasis and inhibition of lipid peroxide accumulation (Yue et al., 2025). Therefore, anti-ferroptotic nanomedicine represents a promising strategy for maintaining chondrocyte viability, interrupting oxidative cell-death cascades, and protecting cartilage from progressive degeneration in OA.

### 4.6 Enhanced synthesis of cartilage matrix components

Ultimately, effective OA therapy requires not only suppressing pathology but also promoting cartilage regeneration. Nanoplatforms facilitate this by delivering chondro-anabolic factors or creating an environment conducive to matrix synthesis, thereby shifting the joint toward an anabolic state. (1) Delivery of chondrogenic factors: Kartogenin (KGN), a small molecule promoting chondrogenesis, is frequently delivered via nanoplatforms to enhance its bioavailability and targeting. CLKM, a hybrid nanoplatform, enhances chondrocyte uptake of KGN and cartilage penetration, promoting cartilage regeneration (Shao et al., 2025). KGN&FP@CV-2, a supramolecular nanomedicine, co-delivers KGN for cartilage regeneration, demonstrating the potential for multi-component delivery (Chen et al., 2024b). PPKHF nanoparticles, encapsulated in hydrogel microspheres, promote chondrocyte differentiation and accelerate cartilage regeneration (Yao et al., 2023). KGN-NPPs (nanocrystal-polymer particles) provide extended release of KGN, improving histological scores and promoting cartilage repair (Maudens et al., 2018). (2) Growth factor and gene delivery: Nanocarriers can protect and deliver sensitive growth factors and genetic material. Dendrimer-based nanocarriers deliver insulin-like growth factor 1 (IGF-1) to chondrocytes, enhancing its therapeutic efficacy and promoting cartilage repair (Geiger et al., 2018). Recombinant adeno-associated virus (rAAV)-mediated co-overexpression of TGF-β and IGF-I enhances chondrogenic differentiation in human mesenchymal stem cells (hMSCs), promoting matrix synthesis (Morscheid et al., 2019). mRNA delivery of the cartilage-anabolic RUNX1 transcription factor via PEG-polyamino acid nanomicelles suppresses OA progression and increases cartilage-anabolic markers, showcasing the precision of gene-level intervention (Aini et al., 2016). (3) Stem cell modulation: Nanomedicines can synergistically enhance the chondrogenesis of mesenchymal stem cells (MSCs) while regulating the inflammatory environment. AHK-CaP/siCA9 NPs, for example, not only repolarize M1 macrophages but also upregulate chondrogenic genes in BMSCs, promoting cartilage repair (Cui et al., 2024). Injectable hydrogel microspheres loaded with PDGF-BB and diclofenac sodium enhance mesenchymal stem cell (MSC) recruitment and chondrogenic differentiation, leading to accelerated cartilage regeneration (Chen et al., 2026). These examples illustrate how nanoplatforms can create a regenerative niche within the joint, fostering both endogenous repair and delivered cell-based therapies.

### 4.7 Cartilage repair effects

The goal of nanoimmunomodulation in OA is to achieve durable cartilage repair and restore joint homeostasis by reprogramming the pathological microenvironment (Chen et al., 2025c; Yao et al., 2025; Yan et al., 2026b). The immune regulatory mechanisms discussed previously translate into tangible cartilage repair effects, which have been demonstrated across various preclinical studies utilizing diverse nanoplatforms and therapeutic strategies. These effects encompass direct protection of existing cartilage, promotion of new cartilage formation, reduction of osteophyte burden, and overall improvement in joint function, providing compelling evidence for the disease-modifying potential of these approaches.

One of the most consistently observed effects is the reduction of cartilage degradation and preservation of cartilage integrity. Nanoplatforms designed to suppress inflammation and oxidative stress directly protect chondrocytes and their surrounding ECM from catabolic processes. For instance, the polycation-functionalized mesoporous ceria nanoparticle (mCNP-G), by scavenging ROS and cfDNA and downregulating pro-inflammatory signaling, significantly reduced osteochondral damage and alleviated symptoms in a rat temporomandibular joint OA model, with histological analysis confirming preserved cartilage structure (Singh et al., 2025). Similarly, black phosphorus nanosheets (BPNSs), through their radical-scavenging and osteogenesis-promoting activities, protected cartilage and prevented OA progression in vivo, with histological and micro-CT analyses confirming their disease-modifying effects by showing reduced cartilage erosion and improved subchondral bone integrity (Lu et al., 2023). The CuMn-ZIF nanozyme, by rebalancing redox status and restoring autophagic flux, improved cartilage and bone health in DMM mice, demonstrating its potent therapeutic effects in mitigating structural damage (Zheng et al., 2026). These examples highlight the direct protective effects of nanoimmunomodulation on cartilage tissue, preventing the irreversible loss characteristic of OA.

Promotion of chondrogenesis and enhanced synthesis of cartilage matrix components are critical for structural cartilage repair in preclinical models, moving beyond mere protection to active regeneration. Nanoplatforms delivering chondro-anabolic factors or creating a pro-regenerative environment are particularly effective in this regard. The metal-organic framework-integrated nanoplatform (CLKM), by delivering kartogenin (KGN) and reprogramming synovial macrophages, preserved cartilage and reduced synovitis in OA mice, leading to restored gait symmetry, indicating functional improvement alongside structural repair (Shao et al., 2025). Exosomes derived from IL-1β-primed human umbilical cord mesenchymal stem cells (C-Exos), especially when encapsulated in hyaluronic acid hydrogel microspheres, enhanced chondrocyte functionality and cartilage matrix production, leading to significant cartilage regeneration in a rat OA model, evidenced by increased proteoglycan and collagen content (Chen et al., 2025a). Chondrocyte-targeting exosomes from umbilical cord-derived MSCs were shown to rejuvenate aging chondrocytes and promote OA cartilage repair in a rat model, suggesting a reversal of age-related cellular dysfunction (Cao et al., 2023). The KGN-loaded biomimetic system (CM@Lipos-KGN) not only mitigated cartilage degeneration and suppressed chondrocyte ferroptosis but also enhanced BMSC chondrogenic differentiation, slowing OA progression and protecting cartilage in animal studies, demonstrating a multi-faceted regenerative effect (Yue et al., 2025). Furthermore, the ultra-long-term anti-inflammatory polyphenol capsule, by remodeling the microenvironment, achieved a single-dose therapeutic effect in mouse and dog OA models, providing an optimal therapeutic window for cartilage repair and highlighting the potential for sustained, long-lasting effects (Wei et al., 2024).

The ability of nanoplatforms to enhance drug delivery and retention within the joint is crucial for these observed repair effects, ensuring that therapeutic agents reach their targets at effective concentrations for sufficient durations. For instance, multi-arm Avidin nano-constructs facilitated intra-cartilage delivery of small molecule drugs like dexamethasone (mAv-Dex), leading to sustained therapeutic doses and suppression of OA-related inflammation and GAG loss better than unmodified dexamethasone, even at low doses (He et al., 2020). This demonstrates that enhanced delivery can significantly improve the efficacy of existing drugs. Cartilage-penetrating cationic peptide carriers (CPCs) demonstrated deep cartilage penetration and retention in a rabbit model of post-traumatic OA, improving intra-joint bioavailability of therapeutics and protecting cartilage, showcasing the importance of overcoming the cartilage barrier (Boyer et al., 2025). The cartilage-targeting and dual MMP-13/pH responsive theranostic nanoprobes (CMFn@HCQ) released hydroxychloroquine (HCQ) in OA joints under acidic pH, reducing synovial inflammation and improving OA outcomes, illustrating the power of smart, responsive delivery systems (Chen et al., 2019; Xi et al., 2026).

Moreover, the synergistic effects achieved by combining different therapeutic modalities within nanoplatforms often lead to superior repair outcomes compared to single-agent approaches. For example, the magnetic polysaccharide hydrogel particles, by encapsulating magnetic nanoparticles and diclofenac sodium, demonstrated synergistic effects in alleviating OA symptoms and promoting cartilage repair, indicating that multi-target interventions are more effective (Yang et al., 2024). Dual-modular hydrogel microparticles (dmHMPs) were specifically designed to modulate the inflammatory microenvironment and dictate full-thickness cartilage regeneration, suppressing Ihh and collagen type X expression while enhancing chondrogenic differentiation, demonstrating a comprehensive approach to guiding cartilage repair (Chen et al., 2025b). The ROS-responsive nanomedicine (G4-TBP NPs-FN) exhibited ROS scavenging, hypoxia attenuation, and M2 macrophage polarization, protecting macrophages and promoting bone repair, ultimately reducing joint pathology and inhibiting synovitis, showcasing how addressing multiple pathological pathways simultaneously leads to superior outcomes (Zhan et al., 2024).

**Fig. 4:**
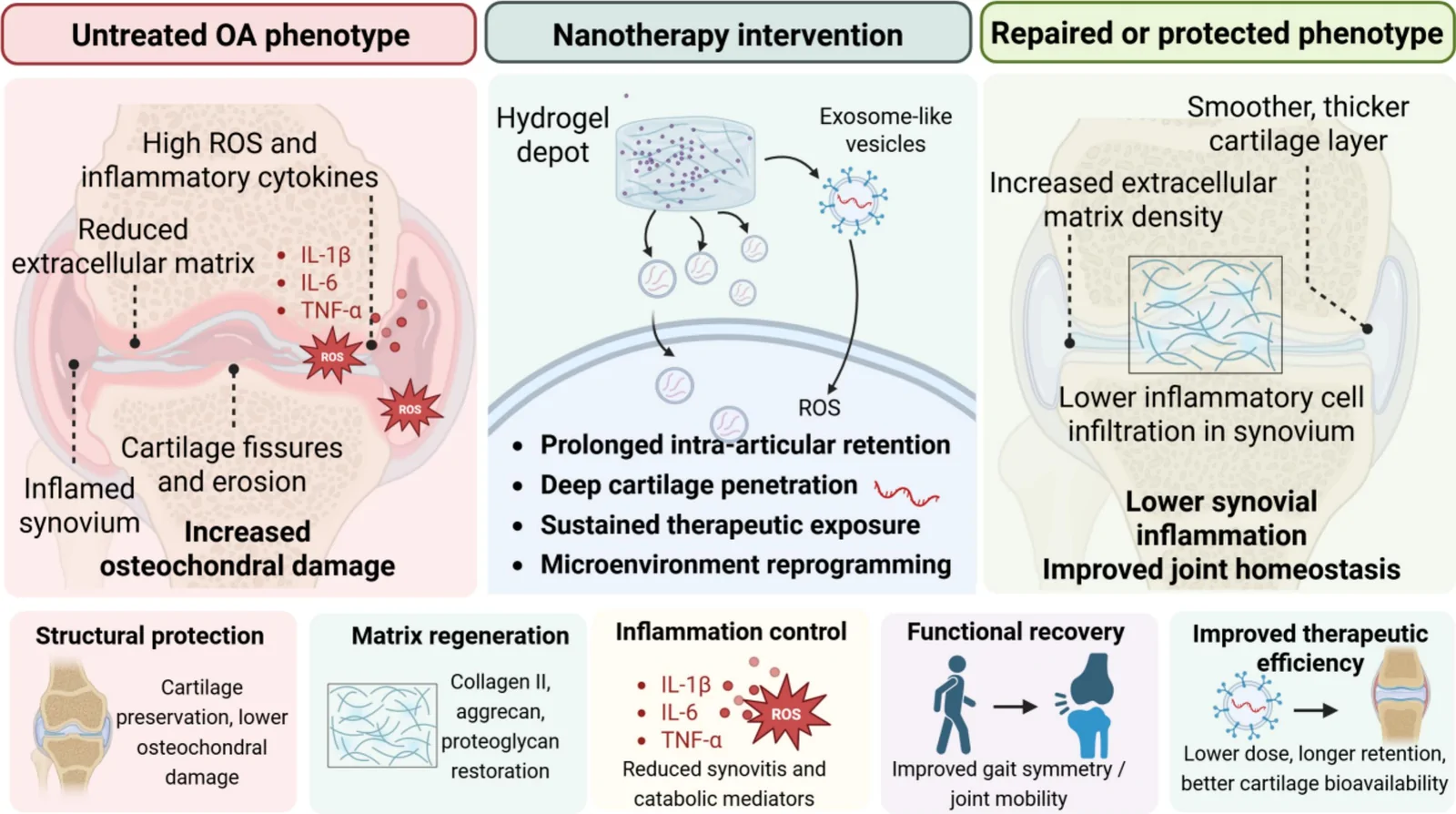
Therapeutic outcomes of nano-immunomodulation for cartilage repair. Nano-immunomodulatory therapies protect cartilage by reducing synovitis, oxidative injury, chondrocyte death, and extracellular matrix breakdown. Their therapeutic effects are reflected by improved cartilage preservation, enhanced proteoglycan and collagen deposition, reduced structural degeneration, and better joint functional recovery.

In summary, nanoimmunomodulation strategies have consistently demonstrated promising cartilage repair effects in preclinical models of OA. These effects stem from their ability to precisely modulate the inflammatory microenvironment, scavenge harmful species, reprogram immune cells, and deliver chondro-anabolic agents in a targeted and sustained manner. The observed outcomes, including reduced cartilage degradation, enhanced matrix synthesis, and improved joint homeostasis, underscore the transformative potential of these approaches for achieving robust structural modification and molecular reprogramming in preclinical OA models (Fig. 4). However, it must be emphasized that these preclinical structural improvements do not yet constitute clinically meaningful disease modification as defined by regulatory agencies.

## 5 Translational challenges

**Fig. 5:**
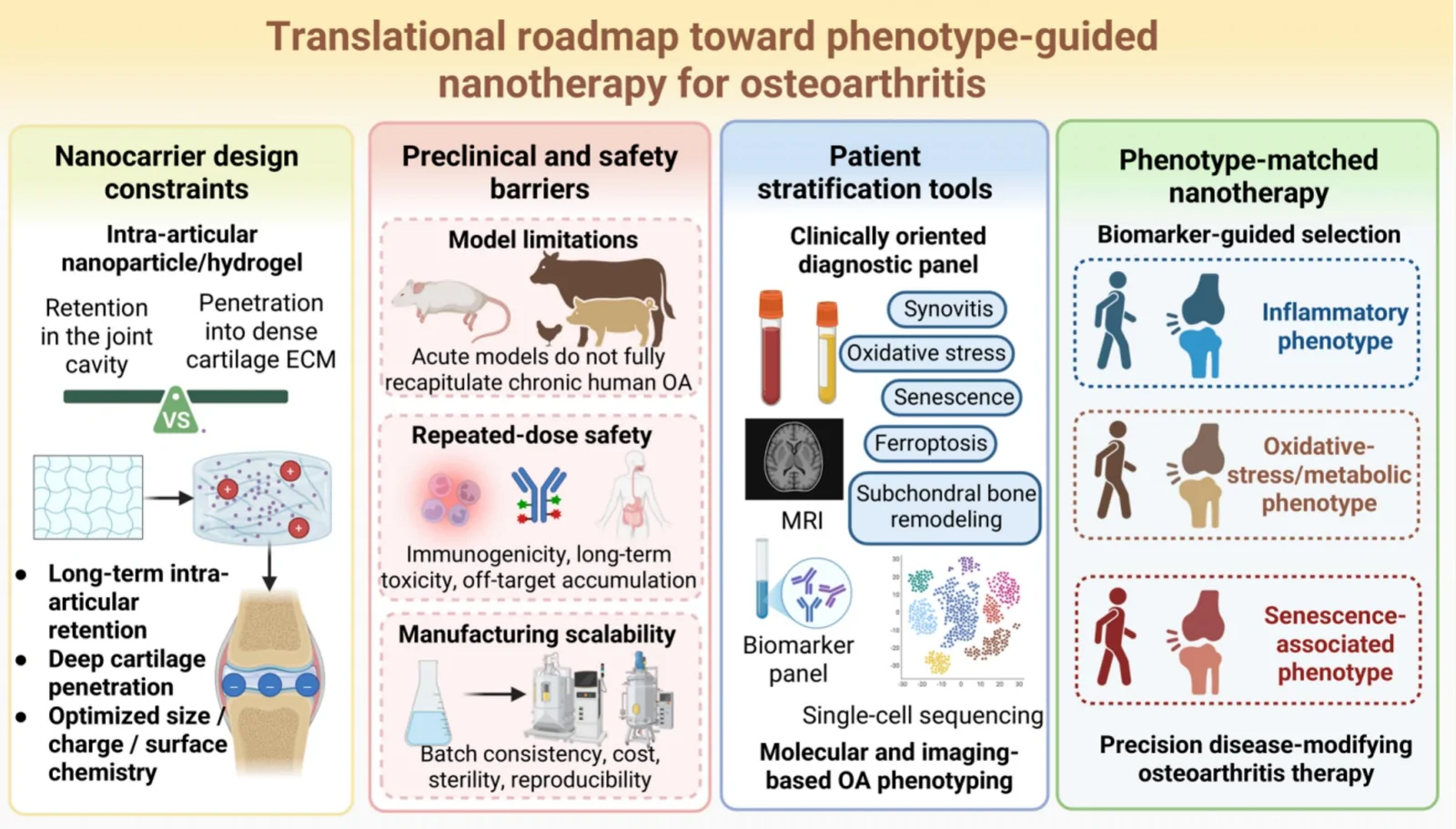
Translational roadmap toward phenotype-guided nanotherapy for osteoarthritis. Clinical translation of osteoarthritis nanotherapy requires balancing intra-articular retention with cartilage penetration while addressing animal model limitations, repeated-dose safety, and scalable manufacturing. Future development should integrate biomarkers, imaging, omics-based patient stratification, and phenotype-matched nanoplatform design to support precision disease-modifying therapy.

In rat model and clinical trials, administering a single intra-articular dose of microsphere-based triamcinolone acetonide (TA) FX006 for knee OA patients resulted in prolonged synovial fluid retention and reduced peak plasma levels, thereby minimizing systemic TA exposure when compared to TA crystalline suspension (TAcs) (Kraus et al., 2018; Kumar et al., 2015). Despite the remarkable progress in nanoimmunomodulation for OA, translating these innovative preclinical findings into clinically viable therapies presents a complex array of challenges. These hurdles span from fundamental scientific limitations to practical manufacturing and regulatory considerations, demanding a concerted effort from researchers, clinicians, and industry stakeholders to bridge the gap between bench and bedside (Fig. 5).

### 5.1 Limitations of animal models

A significant challenge in OA research, particularly for nanomedicine, lies in the inherent limitations of current animal models to fully recapitulate the complexity and chronicity of human OA (Holyoak et al., 2019). Most preclinical studies rely on acute injury-induced models such as the medial meniscus (DMM) mouse model, anterior cruciate ligament transection (ACLT) or chemically-induced monoiodoacetate (MIA) model, which often represent early-stage, post-traumatic OA rather than the chronic, progressive, and multifactorial nature of age-related human OA (Pitcher et al., 2016; Ren et al., 2025). These models may not accurately reflect the long-term inflammatory processes, immune dysregulation, and cellular senescence that characterize human disease (Han et al., 2024). For instance, while a single day of TGF-β1 exposure can activate chondrogenic pathways in bone marrow-derived stromal cells in vitro *(*Futrega et al., *2021**)*, the chronic inflammatory environment in human OA can repurpose such signals, as seen with BMP-2, to drive pathological osteophyte formation rather than repair (Yu et al., 2026). This discrepancy highlights that the pathological context in human OA can fundamentally alter the biological response to therapeutic signals. Furthermore, species-specific differences in joint anatomy, cartilage composition, immune responses, and metabolic profiles can limit the direct translatability of findings from small animal models (mice, rats) to larger animals (rabbits, dogs, horses) and eventually humans (Holyoak et al., 2019). In OA nanotherapy, the core of pharmacokinetics (PK) lies in the spatiotemporal distribution of drugs in the local joint area, rather than the traditional systemic blood drug concentration. If the PK study is only conducted in healthy animal models, it will seriously overestimate the actual retention time under the pathological state of OA. Therefore, the lack of robust, long-term animal models that faithfully mimic human OA progression and its diverse endotypes makes it difficult to predict the long-term efficacy and safety of nanoimmunomodulatory therapies in a clinical setting, particularly regarding chronic effects and potential immunogenicity. Developing more physiologically relevant large animal models that better reflect the human disease progression, and heterogeneity is crucial for validating nanomedicine strategies.

### 5.2 Balance between intra-articular retention and cartilage penetration

Effective intra-articular delivery for OA therapy requires a delicate balance between prolonged retention within the joint space and adequate penetration into the dense, avascular cartilage tissue. These two requirements often present conflicting design parameters for nanocarriers.

#### 5.2.1 Intra-articular retention

Rapid clearance of intra-articularly injected agents is a major impediment to their therapeutic efficacy. Small molecules and even larger proteins are quickly diluted and removed from the joint via lymphatic and vascular drainage, leading to short residence times and necessitating frequent injections (Di Francesco et al., 2022; Kou et al., 2019; Brown et al., 2019). This rapid clearance limits the sustained exposure of therapeutic agents to target tissues, reducing their overall therapeutic impact. To address this, nanoplatforms are engineered to enhance retention. Hydrogel-based systems, for example, provide a sustained release depot within the joint. Injectable hydrogels, such as PEG-4MAL hydrogels, can maintain mechanical properties and release therapeutic payloads over extended periods, protecting the knee joint from cartilage degradation (Holyoak et al., 2019). Similarly, hyaluronic acid-based hydrogels and microgels are widely used for their biocompatibility and ability to prolong drug residence, encapsulating agents like exosomes or drugs for sustained delivery (Chen et al., 2025a; Cao et al., 2023; Lu et al., 2019; Zhang et al., 2016b). Supramolecular nanomedicines, like KGN&FP@CV-2, are designed for sustained drug release and intrinsic lubrication, offering prolonged therapeutic effects (Chen et al., 2024b). Surface modifications of nanoparticles, such as with chondroitin sulfate, can also enhance intra-articular retention by promoting binding to joint tissues, leveraging specific interactions with ECM components (Deng et al., 2025b). The challenge lies in designing systems that are stable enough for long-term retention but also biodegradable and non-toxic.

#### 5.2.2 Cartilage penetration

While retention in the joint space is crucial, therapeutic agents must also penetrate the dense, negatively charged ECM of articular cartilage to reach chondrocytes, which are the primary target cells for cartilage repair. The ECM, rich in negatively charged aggrecans and collagen fibers, acts as a significant barrier to the diffusion of many molecules and nanoparticles, particularly those that are large or negatively charged (He et al., 2020; Vedadghavami et al., 2019). This barrier limits the bioavailability of therapeutics at the site of action within the cartilage. To overcome this, charge-based carriers have been extensively investigated. Cationic nanoparticles and peptides, such as Avidin and cationic peptide carriers (CPCs), leverage electrostatic interactions with the negatively charged aggrecans within the cartilage matrix to facilitate deep penetration and prolonged retention (Vedadghavami et al., 2022; Wang et al., 2026; Han et al., 2026). For instance, multi-arm Avidin nano-constructs demonstrated 112-fold higher uptake in cartilage compared to the equilibration bath and maintained a long residence time via weak-reversible binding with aggrecans, enabling sustained therapeutic doses for weeks (He et al., 2020). Similarly, PEGylated PAMAM dendrimer nanocarriers showed 70% uptake into cartilage tissue and enhanced therapeutic IGF-1 joint residence time by 10-fold for up to 30 days, reducing cartilage degeneration (Geiger et al., 2018). Recent studies confirm that CPCs can penetrate full cartilage depth and be retained for at least one week in a rabbit model of post-traumatic OA, with retention correlating with tissue GAG density (Boyer et al., 2025). The design of these carriers must consider not only charge but also hydrophobicity, as hydrophobic interactions can stabilize intra-cartilage binding in complex synovial fluid environments (Vedadghavami et al., 2022). However, an excessive positive charge can also lead to non-specific binding to other negatively charged components in the synovial fluid, rapid clearance by immune cells, or even induce toxicity to chondrocytes. Therefore, optimizing the charge density, size, and surface chemistry of nanocarriers is critical to achieve both efficient penetration and minimal off-target effects, representing a complex design trade-off.

### 5.3 Safety concerns associated with repeated dosing

The chronic and progressive nature of OA often requires repeated or long-term therapeutic interventions, making safety a central consideration for nanoimmunomodulatory strategies. Unlike short-term treatments, repeated administration of nanomedicines may introduce cumulative risks that are not fully captured in acute efficacy studies. One major concern is immunogenicity, as recurrent exposure to synthetic nanomaterials, biomimetic nanoparticles, or engineered extracellular vesicles may trigger innate or adaptive immune responses, accelerate nanoparticle clearance, reduce therapeutic efficacy, or even induce systemic inflammatory reactions (Chen et al., 2025d). This issue is particularly relevant for complex biologically derived or membrane-coated systems, whose surface components may be recognized by the host immune system after repeated dosing. Another important concern is long-term toxicity. Although many nanoplatforms are designed to be biocompatible or biodegradable, their degradation products, clearance pathways, and potential accumulation within the joint cavity, reticuloendothelial system, or other organs require careful evaluation. This is especially critical for inorganic nanozymes and slowly degradable nanomaterials, which may provide potent antioxidant or immunomodulatory effects but also carry potential risks of bioaccumulation after repeated administration. Its long-term in vivo degradability, heavy metal accumulation toxicity and kidney/liver clearance pathways are the fatal limitations that hinder its clinical transformation (Zhang et al., 2025a). In addition, absolute targeting specificity is difficult to achieve in the complex joint environment, where synovial fluid dynamics, ECM barriers, immune cell uptake, and vascular or lymphatic drainage may contribute to off-target distribution. The sacrificed drug loading capacity resulted in the actual amount of drug reaching the target site being insufficient to produce a lasting therapeutic effect. Repeated exposure could therefore increase the likelihood of unintended tissue accumulation and associated adverse effects. Even materials considered biocompatible may induce low-grade inflammation, foreign-body responses, or synovial irritation over time, potentially aggravating rather than resolving the pathological OA microenvironment. Therefore, before clinical translation, nanoimmunomodulatory therapies must undergo rigorous long-term safety assessment, including repeated-dose toxicology, immunogenicity testing, biodistribution and clearance studies, pharmacokinetic/pharmacodynamic profiling, and evaluation of local joint compatibility. The development of sensitive biomarkers for early detection of immune activation, tissue toxicity, or abnormal nanoparticle accumulation will also be essential for ensuring the safe clinical use of nanomedicines in chronic OA management.

### 5.4 Scalable manufacturing processes

The transition from laboratory-scale synthesis to large-scale, cost-effective, and quality-controlled manufacturing remains a major bottleneck for the clinical translation of nanomedicines, particularly for complex multi-component platforms such as engineered extracellular vesicles, biomimetic nanoparticles, and hydrogel–nanoparticle composites (Liao et al., 2026d, Liao et al., 2026b, Pan et al., 2025, Shi et al., 2025, Wang et al., 2025b). A central challenge is to ensure batch-to-batch consistency in critical quality attributes, including particle size, morphology, surface charge, surface ligand density, drug-loading efficiency, release kinetics, sterility, stability, and biological activity. There is a huge gap between laboratory-level hand-prepared nanoparticles and GMP-level industrial production. Many complex nanostructures that perform well in papers encounter problems such as large batch-to-batch variations, difficult sterilization, and poor stability when scaled up for production (Zhang et al., 2025b; Chen et al., 2025e). Even subtle variations in these parameters may alter biodistribution, intra-articular retention, cartilage penetration, immunomodulatory potency, and safety profiles. This issue is especially pronounced for biologically derived systems such as extracellular vesicles, whose composition and function can be influenced by cell source, culture conditions, isolation methods, and storage procedures (Wu et al., 2026). In addition to reproducibility, manufacturing cost represents another important translational barrier. Many nanomedicine platforms require specialized equipment, high-purity reagents, complex purification processes, and stringent quality-control assays, which may limit affordability and scalability for a chronic and highly prevalent disease such as OA. Emerging manufacturing approaches, including microfluidic synthesis, continuous-flow production, automated purification, and standardized potency assays, may help improve scalability while reducing variability and cost. From a regulatory perspective, OA nanomedicines must also meet rigorous requirements for quality, purity, stability, safety, and efficacy under Good Manufacturing Practice standards, while combination products and biologically engineered systems may face additional uncertainties because their regulatory pathways are still evolving (Shi et al., 2026). Therefore, future development should incorporate manufacturability, quality control, cost-effectiveness, and regulatory feasibility at the early design stage, rather than treating them as downstream considerations after preclinical efficacy has been demonstrated. Establishing robust, reproducible, and scalable manufacturing protocols will be essential for transforming nanoimmunomodulatory strategies from promising experimental platforms into clinically viable and commercially accessible OA therapies.

### 5.5 Patient stratification

OA is not a single, uniform disease entity, but rather a highly heterogeneous syndrome encompassing diverse clinical manifestations, anatomical distributions, molecular drivers, and rates of structural progression Wang et al., 2025a, Xie et al., 2025, Xu et al., 2025a, Zang et al., 2025, Zhu et al., 2026. This heterogeneity represents one of the central barriers to the clinical translation of nanoimmunomodulatory therapies. The future clinical application of nanoimmunomodulation will require a shift from material-centered development toward disease-phenotype-driven therapeutic design, in which nanoplatforms are rationally matched to the specific immune-metabolic state of the diseased joint.

A key prerequisite for this transition is the accurate definition of OA endotypes and phenotypes. Increasing evidence suggests that OA patients can be stratified into biologically meaningful subgroups, such as inflammatory, metabolic, mechanical, pain-dominant, and senescence-associated phenotypes (Karsdal et al., 2025; Saxer et al., 2024). Among these, the inflammatory endotype is particularly relevant to nanoimmunomodulation. Patients with this phenotype may present with synovitis, elevated pro-inflammatory cytokines, activation of innate immune pathways, and increased infiltration or activation of synovial macrophages. In such patients, nanotherapeutic strategies that suppress NF-κB signaling, inhibit inflammasome activation, scavenge inflammatory mediators, or reprogram M1-like macrophages toward pro-resolving M2-like phenotypes may be especially beneficial (Karsdal et al., 2025; Kurz et al., 2025). In contrast, patients with a predominantly oxidative-stress-driven phenotype may respond better to ROS-scavenging nanozymes or mitochondria-targeted antioxidant systems, whereas those with prominent senescence-associated pathology may require senolytic or senomorphic nanomedicines designed to interrupt the SASP. Similarly, patients with mechanically driven OA and limited synovial inflammation may derive less benefit from purely anti-inflammatory nanotherapies unless these are combined with strategies that address cartilage matrix protection, lubrication, or subchondral bone remodeling.

Recent advances in single-cell sequencing and spatial transcriptomics are beginning to reveal the profound cellular and microenvironmental complexity of OA tissues. These technologies allow investigators to map disease-associated chondrocyte subsets, activated synovial fibroblasts, macrophage states, endothelial alterations, and spatially restricted inflammatory niches within the osteoarthritic joint (Xie et al., 2026). Such information is highly relevant for nanomedicine design because it can identify which cell populations should be targeted, which molecular pathways should be modulated, and which anatomical compartments require preferential drug accumulation. For example, if spatial transcriptomic profiling reveals macrophage-rich inflammatory niches within the synovium, a macrophage-targeted nanoplatform may be rationally selected. Conversely, if cartilage zones show dominant oxidative injury and matrix-degrading signatures, cartilage-penetrating antioxidant or anti-catabolic nanoparticles may be more appropriate. Thus, spatially resolved molecular profiling may provide a mechanistic bridge between OA biology and precision nanotherapeutic design.

Building on these spatially resolved molecular insights, the next translational step is to develop clinically applicable biomarkers that can convert tissue-level heterogeneity into actionable patient stratification. Current clinical assessments, including radiographic joint-space narrowing, pain scores, and functional scales, mainly reflect structural damage or symptom burden and cannot adequately define the dominant molecular drivers of OA in individual patients. The biomarker-guided framework would also improve clinical trial design by enriching for biologically responsive subgroups rather than broadly defined OA populations, thereby enhancing statistical power and clarifying mechanisms of action. It is crucial to acknowledge that the concept of matching specific nanoplatforms to OA endotypes remains a theoretical framework rather than a clinically validated practice. While the field recognizes distinct molecular phenotypes, the definitions of these endotypes are largely provisional, derived from post-hoc analyses of clinical trials or cross-sectional cohorts, and lack universally accepted, validated diagnostic cut-offs. Furthermore, no clinical trial has yet demonstrated that biomarker-guided selection of nanotherapies improves outcomes compared to unstratified approaches. Given that OA phenotypes evolve over time, longitudinal biomarker monitoring may further help determine optimal treatment timing, therapeutic adjustment, and biological response. Thus, integrating molecular profiling, biomarker development, and nanoplatform selection will be essential for advancing nanoimmunomodulation from empirical preclinical efficacy toward personalized and clinically translatable disease-modifying therapy.

### 5.6 The regulatory gap: preclinical structural modification vs. clinical disease modification

It is imperative to distinguish between molecular/cellular effects, structural modification observed in preclinical models, and clinically meaningful disease modification. Regulatory agencies FDA and EMA strictly define a DMOAD as an agent that demonstrates a structural effect that translates into, or reliably predicts, a clinically meaningful symptomatic benefit, sustained over time in adequately powered randomized controlled trials. A prominent example is Sprifermin, which improved femorotibial cartilage thickness on MRI in a phase II trial yet failed to demonstrate clear symptomatic benefit (Hochberg et al., 2019). Future nanoimmunomodulatory research must align preclinical endpoints with regulatory expectations, focusing on long-term functional outcomes and validated biomarkers that reliably predict clinical symptom relief, rather than relying solely on short-term histological or imaging-based structural improvements.

## 6 Conclusion

OA, a debilitating whole-joint disease, continues to pose a formidable clinical challenge, with current therapeutic strategies largely confined to symptomatic relief rather than disease modification. The evolving understanding of OA as a complex interplay of inflammation, immune dysregulation, oxidative stress, cellular senescence, and impaired repair mechanisms within a hostile joint microenvironment underscores the urgent need for innovative, disease-modifying interventions. Nanoimmunomodulation has emerged as a highly promising paradigm, offering unprecedented opportunities to precisely target and reprogram this pathological milieu, thereby fostering cartilage repair and restoring joint homeostasis.

Throughout this review, we have elucidated how diverse nanoplatforms—including polymeric nanoparticles, liposomes, inorganic nanozymes, extracellular vesicle-inspired systems, cell-membrane biomimetic nanoparticles, and hydrogel-nano composites—are meticulously engineered to address the multifaceted pathology of OA. These advanced systems leverage their unique physicochemical properties to achieve targeted intra-articular delivery, sustained therapeutic release, and enhanced penetration into the dense cartilage matrix, overcoming the limitations of conventional approaches. The immune regulatory mechanisms mediated by these nanoplatforms are extensive and sophisticated, encompassing the crucial reprogramming of synovial macrophages from a pro-inflammatory M1 to a pro-resolving M2 phenotype, potent suppression of inflammatory signaling pathways, effective scavenging of ROS, and the mitigation of detrimental processes such as cellular senescence and ferroptosis. Critically, these immunomodulatory actions collectively create an environment conducive to the enhanced synthesis of cartilage matrix components, thereby directly contributing to cartilage repair and regeneration. Preclinical studies consistently demonstrate that these nanoimmunomodulatory strategies lead to tangible cartilage repair effects, including reduced cartilage degradation, decreased osteophyte formation, and improved joint function.

However, the journey from promising preclinical results to widespread clinical application is fraught with significant translational challenges. The limitations of current animal models, which often fail to fully recapitulate the chronic and heterogeneous nature of human OA, necessitate the development of more representative preclinical systems. A critical design consideration remains the delicate balance between achieving prolonged intra-articular retention of nanocarriers and ensuring their effective penetration into the avascular, negatively charged cartilage tissue. While innovative strategies like hydrogel encapsulation and charge-based targeting have shown promise in extending residence time and enhancing penetration, optimizing these parameters without compromising safety or inducing off-target effects is paramount. Furthermore, safety concerns associated with repeated dosing, including potential immunogenicity, long-term toxicity, and off-target accumulation, demand rigorous and comprehensive preclinical and clinical evaluation. The scalability and reproducibility of manufacturing processes for complex nanomedicines, particularly engineered extracellular vesicles, represent a substantial hurdle for commercialization and broad accessibility. Finally, the inherent heterogeneity of OA underscores the need for patient stratification, moving beyond a “one-size-fits-all” approach to develop personalized nanotherapeutic strategies tailored to specific disease endotypes or phenotypes.

In conclusion, the field of nanoimmunomodulation for OA is rapidly advancing, offering a transformative vision for disease management. To truly unlock its clinical potential, future research must pivot from a “materials-driven” approach, where novel nanomaterials are developed in isolation, to a more “disease-phenotype-driven” one. This paradigm shift entails a deeper understanding of the specific immune and metabolic phenotypes driving OA in individual patients, followed by the rational design of nanomaterials precisely tailored to address these unique pathological signatures. This will require the integration of advanced diagnostic tools, including spatial transcriptomics and sophisticated imaging, to accurately phenotype the diseased joint microenvironment. By fostering interdisciplinary collaboration and meticulously addressing the translational challenges, we can establish clinically oriented, nanomaterial-based immunotherapeutic strategies that not only alleviate symptoms but also achieve durable cartilage protection and disease modification, ultimately improving the lives of millions affected by OA.

## CRediT authorship contribution statement

**Hanning Wang:** Writing – original draft, Conceptualization. **Fei Wang:** Writing – original draft, Conceptualization. **Sijia Han:** Writing – original draft. **Ling Li:** Data curation, Conceptualization. **Letong Liu:** Writing – review & editing, Methodology. **Zhan Wang:** Writing – review & editing, Methodology.

## Clinical trial number

Not applicable.

## Ethics approval and consent to participate

Not applicable.

## Funding

This research did not receive any specific grant from funding agencies in the public, commercial, or not-for-profit sectors.

## Declaration of competing interest

The authors declare that they have no known competing financial interests or personal relationships that could have appeared to influence the work reported in this paper.

## Data Availability

There is no data associated with this research.
